# Evaluation of the ability of insulin resistance and lipid-related indices to predict the presence of NAFLD in obese adolescents

**DOI:** 10.1186/s12944-024-02144-7

**Published:** 2024-07-02

**Authors:** Aylin Yetim, Memduh Şahin, İbrahim Kandemir, Betül Bulakçı, Melike Tuğrul Aksakal, Edanur Karapınar, Hayrettin Sever, Firdevs Baş

**Affiliations:** 1 Division of Adolescent Medicine, Department of Pediatrics, Faculty of Medicine, Istanbul University Istanbul, Istanbul, Turkey; 2 Adolescent Health PhD Program, Institute of Graduate Studies in Health Sciences, Istanbul University, Istanbul, Turkey; 3 Department of Gastroenterology, University of Health Sciences, Başakşehir Çam and Sakura City Hospital, Başakşehir, İstanbul, Turkey; 4 Department of Pediatrics, Biruni University Faculty of Medicine, Istanbul, Turkey; 5 Department of Family Medicine, Istanbul Prof. Dr. Cemil Tascioglu City Hospital, Istanbul, Turkey; 6 Department of Radiology, Istanbul University Istanbul Faculty of Medicine, Istanbul, Turkey; 7 Department of Pediatric Endocrinology, Istanbul University Istanbul Faculty of Medicine, Istanbul, Turkey

**Keywords:** Adolescent, Obesity, Nonalcoholic fatty liver disease (NAFLD), Triglyceride-glucose indices, Lipid accumulation indices

## Abstract

**Background:**

Nonalcoholic fatty liver disease (NAFLD) has become an important health issue in adolescents. Although several parameters and indices have been investigated for the evaluation of NAFLD in adults, these indices are limited in adolescents. In this study, body mass index, waist circumference, triponderal mass index, HbA1c, homeostatic model assessment insulin resistance (HOMA-IR), triglyceride/high-density lipoprotein (Tg/HDL), the lipid accumulation product (LAP) index, the triglyceride-glucose (TyG) index and the aminotransferase (AT) index were examined together, and their diagnostic values in the clinical treatment of NAFLD were compared.

**Materials and methods:**

Seventynine adolescents (10–19 years old) with obesity who were admitted to a pediatric clinic between January and August 2022 and who were diagnosed with exogenous obesity without any comorbidities were included in the study. The presence of NAFLD was evaluated by liver magnetic resonance imaging. The laboratory findings were obtained retrospectively from system records. Parameters were compared between the NAFLD (+) and NAFLD (-) groups. Logistic regression analysis was used to determine the most effective factors for NAFLD treatment. Receiver operating characteristic (ROC) analysis was performed with significant indices. Sex, HOMA-IR, TyG and AT indices were evaluated together with multivariate analysis to design a diagnostic scale.

**Results:**

HbA1c, HOMA-IR, AT indices and TyG indices were greater in the NAFLD (+) group (*P* = 0.012; *P* = 0.001; *P* = 0.012; *P* = 0.002, respectively). There was a positive correlation between liver fat percentage and HOMA-IR, the TyG index, the AT index, and Tg/HDL. According to the regression analysis, male sex and elevated HOMA-IR were determined to be significant risk factors for the presence of NAFLD. A probability scale with 4 parameters [sex, HOMA-IR, the TyG index, and alanine aminotransferase (ALT)] was designed with 82.5% specificity and 80% sensitivity.

**Conclusion:**

Evaluation of the HOMA-IR and TyG indices, especially in high-risk patients, will support the diagnosis of NAFLD via ultrasonography. A probability scale with ALT, HOMA-IR, TyG, and sex data with a diagnostic accuracy of 80% may aid in the diagnosis of NAFLD in adolescents with obesity.

## Introduction

Childhood obesity is a growing global problem that results in various comorbidities, including nonalcoholic fatty liver disease (NAFLD), which is currently the most prevalent liver disease among children. NAFLD is also recognized as the most common liver disease worldwide. NAFLD is a condition that encompasses a wide spectrum of diseases, from simple steatosis to nonalcoholic steatohepatitis, fibrosis and ultimately cirrhosis and hepatocellular carcinoma. NAFLD is characterized by hepatic steatosis that occurs without any other cause of hepatic fat accumulation, such as excessive alcohol consumption. It is an important cause of cryptogenic cirrhosis [1].

NAFLD is classified into three categories based on histologic findings: (1) NAFLD - fatty liver without hepatocellular damage (> 5% hepatic steatosis); (2) nonalcoholic steatohepatitis (NASH) - fatty liver with hepatocellular damage and inflammation such as hepatocyte ballooning, with or without fibrosis; and (3) cirrhosis with current or previous histologic evidence of NAFLD or nonalcoholic steatohepatitis (NASH) [1].

The pathogenesis of NAFLD has not yet been fully explained. The most widely supported theory is that it is caused by metabolic complications arising from insulin resistance (IR) [2, 3]. Moreover, a new nomenclature has been used in the literature due to the metabolic disorders associated with the pathogenesis of NAFLD, metabolic (dysfunction)-associated fatty liver disease (MAFLD). MAFLD is defined as one or more criteria accompanying fatty liver, overweight/obesity, type 2 diabetes, or two or more signs of metabolic dysfunction [4]. Additionally, there are NAFLD patients in whom metabolic problems are not prominent but genetic predispositions play an active role; this condition is called genetically acquired fatty liver disease (GAFLD) [5]. The genes most strongly associated with the development of NAFLD in the literature are patatin-like phospholipase domain containing protein 3 (PNPLA3) and transmembrane 6 superfamily member 2 (TM6SF2). A meta-analysis showed that PNPLA3 with a homozygous mutant G allele variant was closely associated with hepatosteatosis and its severity [6]. The PNPLPA3 rs738409 gene polymorphism is commonly associated with poor metabolic status, increased body mass index (BMI), dyslipidemia and insulin resistance (IR) in NAFLD patients [7]. A recent animal study revealed that TM6SF2 deficiency reduced lipidation but not the transport of very low-density lipoprotein (VLDL) in hepatocytes [8]. Additionally, a recent review indicated that the TM6SF2 E167K variant is associated with NAFLD in children and adolescents [9].

NAFLD is a hepatic marker of metabolic syndrome (MetS). Elevated serum triglyceride (Tg) and fasting plasma glucose (FBG) levels, which are the most prominent indicators of MetS, play a key role in the development of fatty liver [3, 10]. Therefore, measurements and indices related to MetS are being extensively researched within the context of NAFLD diagnosis. Two commonly used indices are the homeostatic model assessment of insulin resistance (HOMA-IR) score and triglyceride-glucose (TyG) index. Zhang et al. [11] conducted a study on TyG indices and alanine transaminase (ALT) levels in adults with hepatosteatosis and found that the TyG index was an important diagnostic tool for NAFLD and specified a cutoff value. Another index researched in this field is the lipid accumulation product (LAP) index. Recent articles suggest that the LAP index can be used as an indicator of type 2 diabetes (T2D), IR, MetS and NAFLD in adults. Additionally, LAP indices may be associated with the risk of cardiovascular diseases [12–15].

The tri-ponderal mass index (TPMI) has been accepted as a more accurate method of fat quantification than the body mass index (BMI) and BMI standard deviation score (SDS) in the general pediatric population [16]. Basarır et al. compared the TPMI with BMI and BMI SDS values and evaluated it for its ability to support the diagnosis of NAFLD [17].

In the literature, there are studies investigating various indices calculated with clinical and laboratory parameters, as well as imaging modalities for the diagnosis of NAFLD in adults [10–19]. However, similar studies in the adolescent population are limited. The aim of this study was to examine several clinical and laboratory parameters, together with the indices calculated with these parameters, to compare their diagnostic values using magnetic resonance imaging (MRI) in a specific group of adolescents with obesity with/without NAFLD and to develop a probability scale that clinicians can easily use in their routine clinical practices in the diagnosis of NAFLD.

## Materials and methods

This study included adolescents who were diagnosed with exogenous obesity at the Adolescent Health Outpatient Clinic of Istanbul University Faculty of Medicine. Adolescents between the ages of 10 and 19 who visited our clinic between January and August 2022 and had a BMI greater than the 95th percentile for their age and sex were included. Patients with chronic diseases, regular medication use and liver/heart/kidney/thyroid diseases were excluded. A power analysis was performed to determine the number of patients required for the study, and it is estimated that 34 patients in each group are needed to detect a difference (with > 90% probability and δ ≥ 0.8 effect size), assuming a two-sided criterion for detection that allows for a maximum Type I error rate of a = 0.05. More patients were included in the study due to data loss (a total of 79 adolescents with obesity, 39 without NAFLD and 40 with NAFLD).

Clinical assessments and measurements were performed by an experienced pediatrician. A wall-mounted, calibrated Harpenden stadiometer (Holtain Ltd., Crymych, UK) and an electronic scale (sensitivity of 0.1 kg) were used. BMI was calculated in kg/m^2,^ and the TPMI was calculated in kg/m^3^. Waist circumference (WC) was measured at the midpoint between the lower edge of the last palpable rib and the top of the iliac crest using inextensible tape. Standard deviation (SDS) calculations of weight, height, BMI and WC were performed according to national data [20, 21]. The 12-hour fasting blood samples of adolescents with obesity who were examined in our adolescent outpatient clinic were evaluated for laboratory analysis. The laboratory values (FBG, insulin, hemoglobin A1c (HbA1c), alanine aminotransferase (ALT), aspartate aminotransferase (AST), gamma glutamyl transferase (GGT), albumin, total cholesterol, triglyceride (Tg), low density lipoprotein (LDL), high density lipoprotein (HDL), C-reactive protein (CRP), and platelets) of the included patients were obtained retrospectively from the records.

HOMA-IR was calculated using the following formula: FBGxInsulin/405. The lipid accumulation index was calculated as [WC (cm)-65] x Tg (mmol/L) for boys and [WC (cm)-58] x Tg (mmol/L) for girls. The aminotransferase (AT) index was calculated as ALT/AST, and the TyG index was calculated as Ln[Tg(mg/dl) xFBG(mg/dL)/2].

Liver MRI was conducted using a 1.5 Tesla (T) system (Magnetom Aera; Siemens Healthineers, Erlangen, Germany) equipped with an 18-channel body matrix coil and a 32-channel spine matrix coil. Scans were performed without sedation/anesthesia or the use of contrast agents. Proton density fat fraction measurements were taken from the fat fraction map generated by the MRI system console. Patients with an MRI value of 5 or greater on liver MRI were classified as having NAFLD, and those with an MRI value of less than 5 were classified as not having NAFLD.

Ethics Committee approval (No. 604,312) was obtained from the Istanbul University Faculty of Medicine, and informed consent was obtained from all patients and their families.

### Statistical analysis

All analyses were performed using SPSS version 21.0 for Windows (IBM, Inc., Chicago, Illinois, USA) and JAMOVI 2.3.18 with the GamLj and psychoPDA extensions. Kurtosis and skewness values between + 2 and − 2 were considered to indicate a normal distribution, and parameters outside these limits indicated a nonnormal distribution. Differences between groups were analyzed by independent samples t tests for homogeneous groups and Mann‒Whitney U tests for nonhomogeneous groups. Correlations were analyzed using Pearson and Spearman analyses. Analysis results with a *P* value < 0.05 were considered significant.

In the multivariate logistic regression analysis (logistic regression and generalized linear model), the dependent variable was the presence of NAFLD, and the independent variables were sex, HOMA-IR, the TyG index, and the AT index. The cutoff values were determined by receiver operating characteristic (ROC) analysis for the HOMA-IR and TyG indices, which are strongly associated with NAFLD according to independent sample t tests and correlation analysis.

The probability scale for NAFLD diagnosis was built with the best positive predictive values and specificity cutoff values. ROC analysis of ALT, AT indices, FBG, insulin, HbA1c, HOMA-IR, TyG indices, LAP indices, Tg/HDL, sex and all areas under the curve (AUCs) > 0.7 was performed. The best positive predictive values were selected as the threshold, and then a primitive NAFLD probability scale was built by setting 0 (< threshold or null) or 1 (≥ threshold or positive) for the laboratory results of ALT, TyG and HOMA-IR levels. Sex was also added to the scale because it was correlated with NAFLD. The ALT threshold was 22 (ALT; ≤22 = 0, > 22 = 1), the TyG index was 8.38 (TyG; ≤ 8.38 = 0, > 8.38 = 1), and the HOMA-IR index was 6.3 (HOMA-IR; ≤6.3 = 0, > 6.3 = 1) for the best positive predictive values. Scoring in terms of gender factors was performed as follows: male gender = 1, female gender = 0. It should be emphasized that to create a probability scale, the thresholds in this scale focus on positive predictive values and specificity results. The threshold of 4.55 for HOMA-IR was the best for mutual specificity and sensitivity. However, in this scale, a value of 4.55 was not considered; instead, a value of 6.3 was considered to ensure specificity and the best positive predictive value. The minimum result was considered to be 0, and the maximum was 4.

## Results

The average age of the patients, consisting of 39 females and 40 males, was 14.5 ± 2.2 years (range 10–19). The mean ages of the groups were similar. A chi-square test was performed to compare genders between the groups, revealing a *P* value of *P* = 0.007 (boys 65% girls 35%).

Table 1 presents the demographic characteristics and laboratory values of NAFLD (+) and NAFLD (-) patients. The NAFLD (+) group had significantly greater AT indices, Tg levels, TyG indices, FBG levels, insulin levels, HbA1c levels and HOMA-IR levels than did the NAFLD (-) group (*P* = 0.012; *P* = 0.049; *P* = 0.002; *P* = 0.013; *P* = 0.02; *P* = 0.012; *P* = 0.001, respectively). No significant difference was found between the groups in terms of BMI SDS, TPMI, the Tg/HDL ratio or the LAP index.

**Table 1:** Comparison of Physical and Laboratory Measurements and Indices Used to Diagnose NAFLD in Adolescents with Obesity

Parameter	Total *n* = 79		NAFLD (-) *n* = 39		NAFLD (+) *n* = 40		*P*
	Mean ± SD	Min–max	Mean ± SD	Min–max	Mean ± SD	Min–max	
**Age (Year)**	14.5 ± 2.2	10–18	14.7 ± 2.2	10–18	14.2 ± 2.2	10–18	0.299
**BMI SDS**	2.7 ± 0.65	1.7–4.5	2.8 ± 0.6	2–4.2	2.6 ± 0.7	1.7–4.5	0.446
**WC SDS**	3.2 ± 0.9	0.3–4.9	3.2 ± 0.9	0.3–4.5	3.2 ± 1	1.3–4.9	0.901
**TPMI**	19.9 ± 3.1	16.2–32.5	19.7 ± 2.4	16.6–28.2	20.2 ± 3.7	16.2–32.5	0.428
**Platelet(x10** ^**3**^ **)**	314.2 ± 72.7	157–482	298.6 ± 74.7	157–466	329.4 ± 68.2	197–482	0.59
**CRP (mg/L)**	2.4 ± 1.7	0.1–9	2.1 ± 1.1	0.1–6.2	2.7 ± 2.1	0.3–9	0.105
**ALT (IU/L)**	25.1 ± 16.09	8–104	20.1 ± 11.9	8–62	30 ± 18.2	14–104	0.05
**AST (IU/L)**	22.1 ± 9.4	11–62	19.6 ± 8.5	11–62	24.5 ± 9.7	14–60	0.18
**AT indice (ALT/AST)**	1.1 ± 0.3	0.5–2.1	1 ± 0.3	0.5–1.7	1.2 ± 0.3	0.7–2.1	**0.012**
**GGT (IU/L)**	17.8 ± 10.6	0.34–72	15.9 ± 8.7	9–45	19.5 ± 12.0	0.34–72	0.13
**Albumin (g/dL)**	4.8 ± 0.3	4–5.3	4.74 ± 0.23	4.4–5.2	4.8 ± 0.3	4–5.3	0.14
**Total** **Cholesterol (mg/dL)**	167 ± 31.6	115–276	167.3 ± 29.8	126–276	166.8 ± 33.7	115–258	0.94
**Tg (mg/dL)**	115.5 ± 51.8	41–276	103.8 ± 51.7	45–276	127 ± 49.9	41–265	**0.049**
**HDL (mg/dL)**	45.2 ± 9.9	29.4–75	47.2 ± 9.52	29.4–65	43.2 ± 9.9	30–75	0.68
**Tg/HDL**	2.8 ± 1.8	0.7–9.4	2.5 ± 1.8	0.9–9.4	3.2 ± 1.7	0.7–8	0.73
**FBG (mg/dL)**	88.1 ± 12.9	19–140	84.4 ± 13.1	19–101	91.6 ± 11.9	77–140	**0.013**
**İnsulin (IU)**	23.4 ± 13.5	4.9–66.3	18.7 ± 10.8	4.9–57	28.2 ± 14.4	7.1–66.3	**0.02**
**HbA1c (%)**	5.4 ± 0.4	4.6–6.4	5.26 ± 0.36	4.6–6.2	5.5 ± 0.4	4.7–6.4	**0.012**
**HOMA-IR**	5.2 ± 3.4	0.9–19.4	3.9 ± 2.3	0.9–12.2	6.5 ± 3.8	1.4–19.4	**0.001**
**TyG indice**	8.4 ± 0.5	6.8–9.4	8.3 ± 0.5	6.8–9.4	8.6 ± 0.4	7.4–9.3	**0.002**
**LAP indice**	47.5 ± 29.7	7.6–143.5	42.4 ± 27	7.6–143.5	52.6 ± 31.8	8.14–134	0.131

>BMI SDS = body mass index-standard deviation score; WC SDS = waist circumference-standard deviation score; TPMI = triponderal mass index; LAP index = lipid accumulation product index; AT index = aminotransferaz index; TyG index = triglyceride-glucose index; HOMA-IR = homeostatic model assessment insulin resistance; CRP = C-reactive protein; ALT = alanine aminotransferase; AST = aspartate aminotransferase; Tg = triglyceride; HDL = high-density lipoprotein; FBG = fasting plasma glucose

In the NAFLD (+) group, significant positive correlations were found between HOMA-IR, TyG, AT indices and Tg/HDL indices and the percentage of fatty deposits determined by liver MRI (Table 2). Furthermore, 48.1% (*n* = 38) of patients in both groups had elevated Tg levels (≥ 100 mg/dl) [63.2% (*n* = 24) in the NAFLD (+) group and 36.8% (*n* = 14) in the NAFLD (-) group had elevated Tg levels, *P* = 0.018].

**Table 2:** Correlation analysis of NAFLD indices and MRI data

	MRI-Spect	HOMA-IR	TyG indice	AT indice	LAP indice	Tg/HDL
MRI-Spect						
r	1	0.48**	0.31^**^	0.33^**^	0.151	0.228^*^
*P*		0.001	0.005	0.003	0.187	0.045
HOMA-IR						
r	0.48**	1	0.32**	0.41**	0.185	0.162
*P*	0.001		0.005	0.001	0.108	0.160
TyG indice						
r	0.31^**^	0.32**	1	0.33^**^	0.665**	0.821**
*P*	0.005	0.005		0.003	0.001	0.001
AT indice						
r	0.33**	0.41**	0.33**	1	0.271*	0.246*
*P*	0.003	0.001	0.003		0.016	0.03
LAP indice						
r	0.151	0.185	0.665**	0.271*	1	0.793**
*P*	0.187	0.108	0.001	0.016		0.001
Tg/HDL						
r	0.228*	0.162	0.821**	0.246*	0.793*	1
*P*	0.045	0.160	0.001	0.030	0.001	

** Corelation is significant at the 0.01 level, * Corelation is significant at the 0.05 level

MRI-Spect = magnetic resonance imaging spectroscopy; LAP index = lipid accumulation product index; AT index = aminotransferaz index; TyG index = triglyceride-glucose index; HOMA-IR = homeostatic model assessment insulin resistance, Tg = triglyceride; HDL = high-density lipoprotein

In the multivariate logistic regression analysis performed to determine the factors most effective in the diagnosis of NAFLD, male sex and HOMA-IR level were found to be significant risk factors for the presence of NAFLD (Table 3).

**Table 3:** Evaluation of the Parameters Affecting NAFLD in Obese Adolescents via Logistic Regression Analysis

	B	SE	*P*	ExpB	95% confidence interval	
					Lower	Upper
**Male Gender**	1.233	0.538	0.022	3.436	1.195	9.901
**HOMA-IR**	0.315	0.113	0.005	1.371	1.097	1.712
**AT index**	1.689	0.988	0.087	5.415	0.781	37.57

HOMA-IR = Homeostatic Model Assessment Insulin Resistance; AT index = Aminotransferaz index

**Fig. 1:**
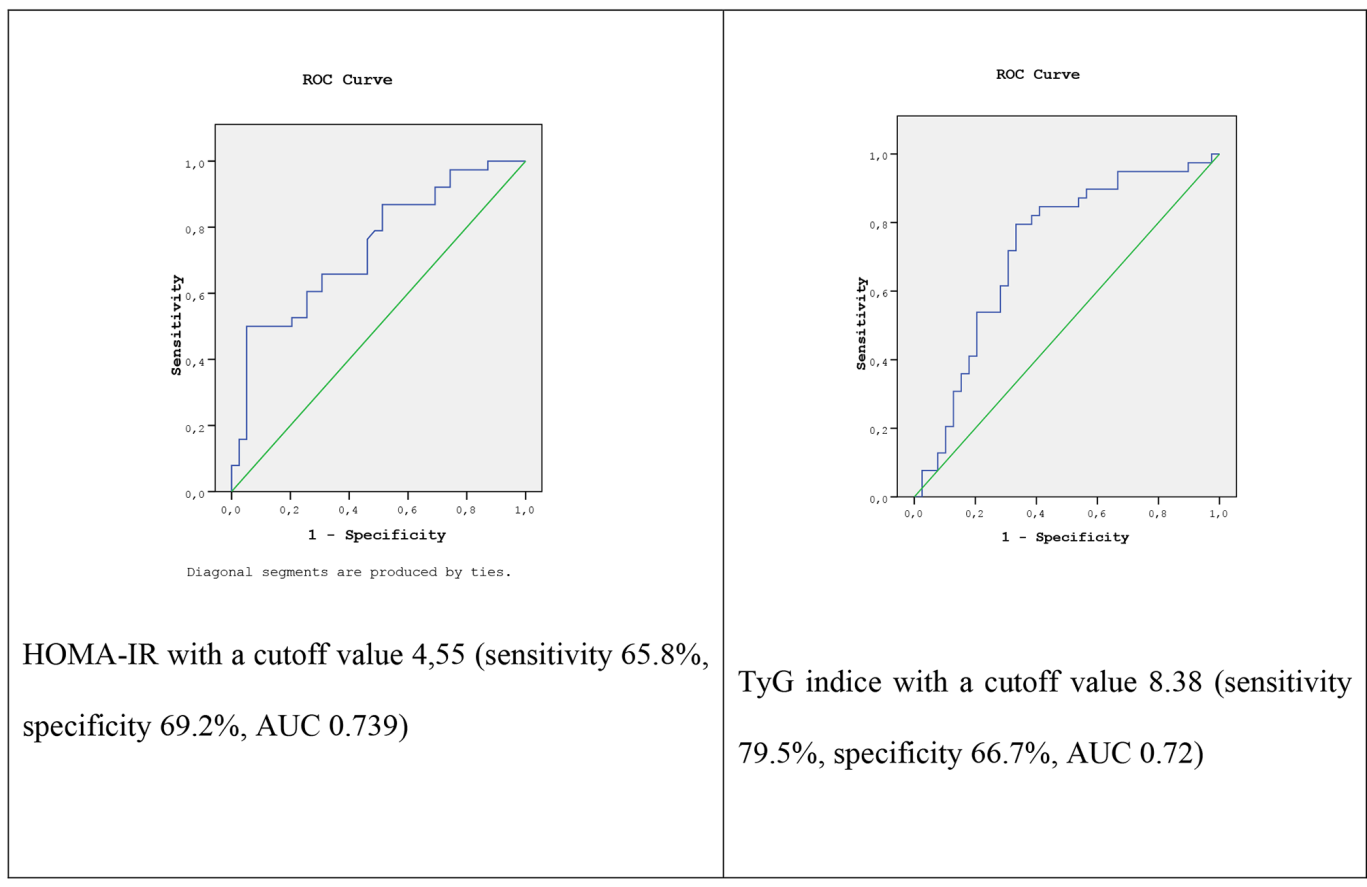
ROC curve analysis for HOMA-IR and the TyG index in NAFLD diagnosis

Since the HOMA-IR and TyG indices were significantly different in the NAFLD (+) group and were strongly correlated with the amount of MRI fat in the liver, ROC curve analysis was performed using the HOMA-IR and TyG indices for the diagnosis of NAFLD. ROC curve analysis of HOMA-IR revealed a cutoff value of 4.55 (*P* < 0.001), an AUC of 0.739, a sensitivity of 65.8%, and a specificity of 68.4%. ROC curve analysis of the TyG index revealed a cutoff value of 8.38 (*P* = 0.001), an AUC of 0.72, a sensitivity of 79.5% and a specificity of 66.7% (Fig. 1). The best threshold value for specificity (67.5%) and sensitivity (67.5%) was 1.07, but the AT index did not provide a powerful result in the ROC analysis (AUC = 0.682).

**Fig. 2:**
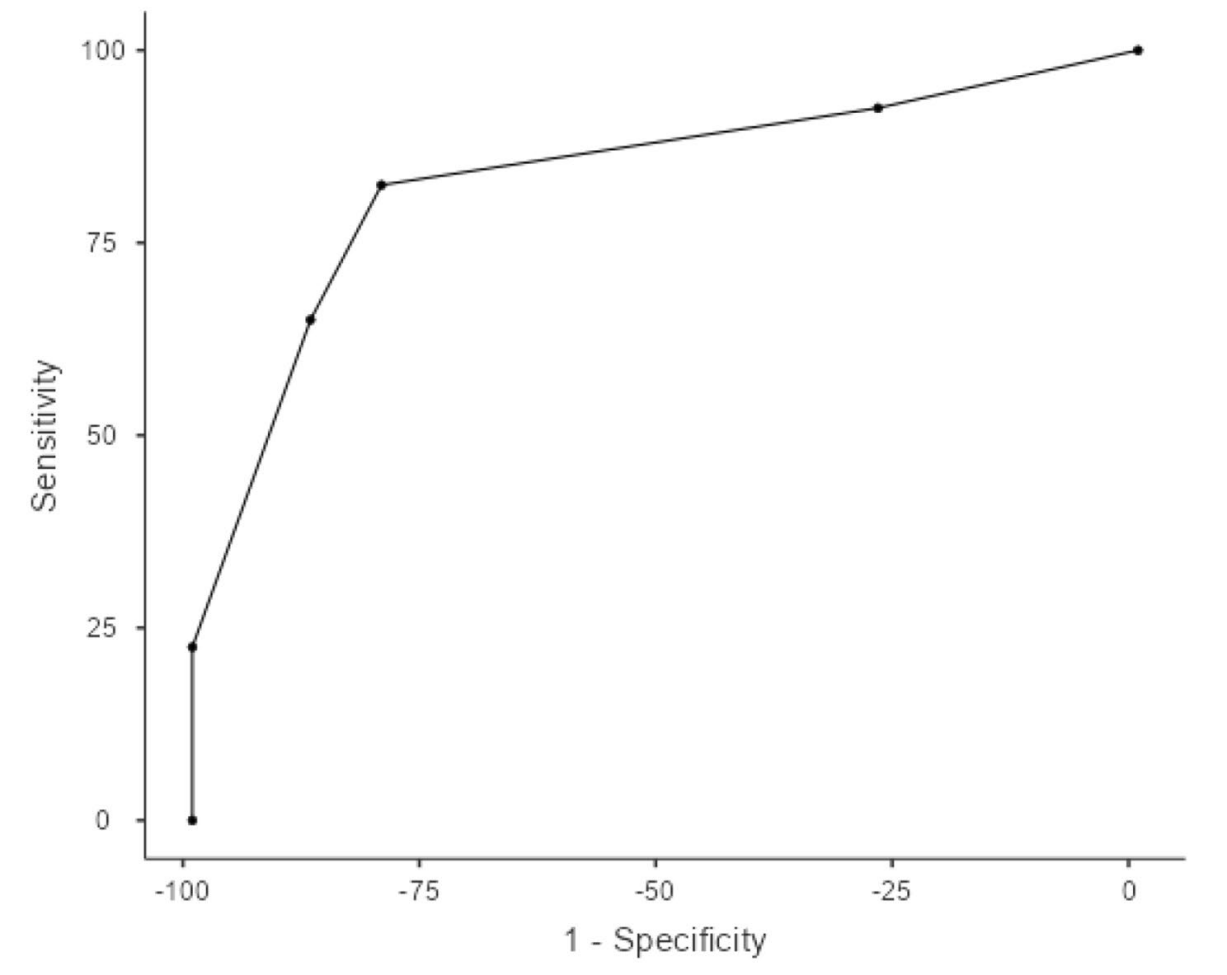
ROC analysis graph built with the designed NAFLD probability scale

The probability scale, consisting of four components (sex, ALT, TyG and HOMA-IR), had a positive predictive value of 80%, a specificity of 82.5%, a sensitivity of 80% and a cutoff point of 3. The AUC was 0.834 (Fig. 2).

**Fig. 3:**
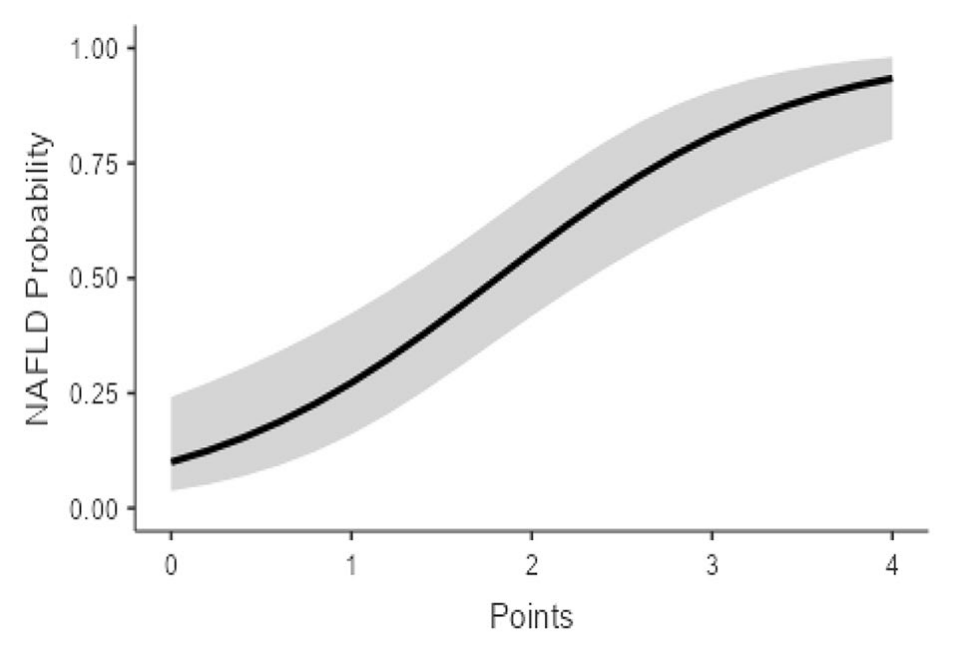
Estimated marginal means graph regarding NAFLD in relation to the designed NAFLD probability scale (Note. The gray zone indicates the 95% confidence interval)

According to the multivariate analysis, each point increased the NAFLD risk by 3.36 times (95% CI: 2.11–5.88) (generalized linear model, R^2^:0.297, loglikelihood ratio test for points (X^2^):33, *P* < 0.001) (Fig. 3; Table 4).

**Table 4:** Estimated mean probabilities of NAFLD according to points in the chart

Points	NAFLD Probablity (95% CI)
0	10.0% (3.8–24.2)
1	27.3% (16.0-42.4)
2	55.7% (41.8–68.9)
3	80.9% (64.8–90.7)
4	93.4% (80.1–98.1)

CI: Confidence interval

## Discussion

In this study, the effectiveness of clinical and laboratory data and indices calculated with the same parameters for the diagnosis of NAFLD in adolescents with obesity were compared between NAFLD groups. The BMI SDS, WC SDS, TPMI, HbA1c, HOMA-IR, Tg/HDL, LAP index, TyG index, and AT index were evaluated together. The TyG index and HOMA-IR were found to be the most useful tools, and cutoff values of 8.38 and 4.55, respectively, were considered warning signs of NAFLD in adolescents with obesity. In addition, AT indices, HbA1c and male sex were significantly greater in the NAFLD (+) group. In light of these data, a probability scale with 80% sensitivity and 82.5% specificity (including sex, ALT, HOMA-IR, and TyG) that can be used in the diagnosis of NAFLD was proposed.

Although the relationship between NAFLD and these indices has been previously studied in the literature, studies on this topic in adolescents are limited [11–19]. Song et al. [22] investigated the TyG index and modified TyG index in adolescent NAFLD patients and reported that the indices were significantly greater in adolescent NAFLD patients than in healthy individuals, which is in line with the severity of steatosis. Ye et al. [23] investigated pentraxin-3 and the TyG index in children and found that the TyG index was a statistically significant predictor of NAFLD, with a cutoff value of 8.16. In this study, in line with these studies, the TyG index in NAFLD (+) adolescents with obesity was found to be significantly greater than that in NAFLD (-) adolescents, and the cutoff value was 8.38. In a cohort study conducted with adult NAFLD patients, 8.5 was recommended as the cutoff value for the TyG index for the diagnosis of NAFLD [11]. Although studies in children and adolescents are limited, according to the literature, the TyG index can be considered a cost-effective tool that can be used in the diagnosis of NAFLD and does not require additional examination. Studies have shown that the cutoff value of the TyG index for the diagnosis of NAFLD is between 8 and 8.5.

Another index researched for NAFLD diagnosis is the LAP index. A meta-analysis revealed that the LAP index could serve as a cost-effective and practical diagnostic tool for NAFLD patients [24]. They evaluated data from 16 studies, and a significant increase in the LAP index among NAFLD patients was found in 14 studies. However, it is important to note that most of these studies focused on adults with NAFLD diagnosed by ultrasonography (US) as part of general population screening. Many factors can contribute to the development of NAFLD in adults. However, the etiology of NAFLD in children and adolescents is less complex, and such markers can be evaluated more meaningfully. In a rare study conducted in obese children aged 6–18 years, the LAP index was found to be significant for the diagnosis of NAFLD, with a cutoff value of 42.7 (sensitivity 53.7%, specificity 84.6%) [25]. Dai et al. [26] presented the results of a cohort study with a total of 40,459 adult participants and demonstrated its usefulness in the diagnosis of NAFLD with a cutoff value of 30.5 for men (sensitivity: 77%, specificity: 75%) and 23.0 for women (sensitivity: 82%, specificity: 79%). This study is the first to examine the usefulness of the LAP index in the diagnosis of NAFLD in adolescent patients with obesity alone by determining liver fat percentage using liver MRI. Although the LAP value was greater in the NAFLD (+) group, the difference was not statistically significant. However, a positive correlation was found between the LAP index and the fat percentage of the liver. More research and studies with larger patient groups are needed to support the use of the LAP index in adolescent NAFLD patients.

Fatty acids accumulate in the liver via hepatocellular uptake from plasma and de novo biosynthesis and are eliminated intracellularly by oxidation or by the secretion of Tg-rich very low-density lipoproteins into the plasma. In cases of overnutrition and obesity, hepatic fatty acid metabolism is altered, often leading to the accumulation of Tg in hepatocytes and NAFLD [27]. Although the role of Tg in the mechanism of NAFLD development is at the forefront, the use of Tg alone is not recommended in the literature, and indices that include Tg, such as the TyG and LAP indices, are available [11, 22–26]. In this study, Tg levels (≥ 100 mg/dl) were found to be approximately twice as high in NAFLD (+) adolescents with obesity than in NAFLD (-) adolescents. However, using Tg in complex indices such as TyG can provide more valuable data.

The TPMI is an index produced as an alternative to BMI and can be used to determine body fat during adolescence without requiring different threshold values according to many variables [28]. It has also been shown to be associated with insulin resistance in children and adolescents [29]. Along with these features, the relationship between the TPMI and NAFLD in children with obesity (6–18 years old) was evaluated by Basarir et al. [17], and its superiority over BMI was not shown. However, they found both BMI and TPMI to be greater in the NAFLD (+) obese group than in the NAFLD (-) group. In this study, no relationships were found between BMI, BMI SDS, TPMI or other anthropometric indices and the presence of NAFLD. The average age of the total patient group was 11.5 years in Basarir et al.’s study [17], while it was 14.5 years in the present study. In addition, while the average age of both groups was similar and consisted mostly of pubertal patients, Basarir et al.‘s study [17] revealed that the average age in the NAFLD (+) group was greater than that in the other group and that there were more prepubertal patients in the NAFLD (-) group. The different findings may be due to the different populations included in the studies. The waist‒hip ratio, which Umano et al. [30] found to be significant, may be related to NAFLD. Additionally, the use of modified TyG indices suggested by Song et al. [22] should also be evaluated in the adolescent population, as their relationship with NAFLD has been shown.

Do Nascimento et al. [31] compared NAFLD (+) and NAFLD (-) adolescents diagnosed with NAFLD by MRI and found a statistically significant difference in terms of HOMA-IR values. The authors reported average HOMA-IR values of 3.48 in the NAFLD (+) group and 1.2 in the NAFLD (-) group and 3.47 in the obese group and 1.21 in the nonobese group. Similarly, in the comparison of HOMA-IR levels between the obese NAFLD (+) and NAFLD (-) adolescent groups in this study, HOMA-IR was found to be significantly greater in the obese NAFLD (+) group. Do Nascimento et al. [31] diagnosed NAFLD with MRI, similar to the current study, but reported the results of their study with a total of 50 participants, half of whom were adolescents of a healthy weight. In this study, the values of a total of 79 adolescents with obesity were compared, and the average HOMA-IR was found to be 6.5. According to the regression analysis, the HOMA-IR value was identified as one of the most significant factors for the presence of NAFLD. This finding indicates that insulin resistance is the primary factor in the development of NAFLD in adolescents, as stated in the literature.

In the study by Nobili et al. [32] in which NAFLD was diagnosed by biopsy, they found no difference in the HbA1c values between the NAFLD (+) and NAFLD (-) groups. However, with respect to glucose tolerance, HbA1c was significantly greater in the NAFLD (+) group [32]. Similarly, in this study, HbA1c values were significantly greater in NAFLD (+) adolescents with obesity than in NAFLD (-) adolescents. However, whether the participants were prediabetic was not considered. The results suggest that the combination of obesity and NAFLD may increase HbA1c values independently of other factors associated with diabetes.

Most studies on NAFLD show a greater prevalence in the male population than in the female population. A recent meta-analysis evaluating 72 studies revealed that it is 2.4 times more common in males [33]. Similarly, Villanueva-Ortega et al. [34] investigated sex-specific differences in NAFLD in the pediatric population, which included individuals who were eutrophic, overweight and obese, and found that the frequency of NAFLD was 2 times greater in boys. In a comprehensive meta-analysis examining the prevalence of NAFLD in the 1–19 age range, it was shown that the frequency of NAFLD was greater in boys, regardless of the diagnostic method [35]. The current study was conducted on adolescents with obesity, and, similar to the literature, the frequency of NAFLD was found to be approximately twice as high in males.

In the adolescent population, an ALT level above 50 IU/L in boys and above 44 IU/L in girls has been reported to have 88% sensitivity but only 26% specificity for diagnosing NAFLD [36]. Additionally, 85% of obese individuals with high ALT values have NAFLD [37, 38]. In this study, the ALT level was found to have borderline significance in the diagnosis of NAFLD. Although it is known that elevated ALT is important for NAFLD diagnosis, it is insufficient to confirm histological findings [39]. Lu et al. [40] conducted a study investigating AT indices in children and adolescents diagnosed with NAFLD (+) by US. The study revealed that participants with a greater AT index had a 2.19-fold greater risk of developing NAFLD (95% CI: 1.24–3.87; *P* = 0.007). In this study, adolescents with obesity diagnosed with NAFLD by MRI were examined, and AT indices, but not ALT or AST indices, were significantly greater in the NAFLD (+) group than in the NAFLD (-) group. Additionally, the AT index was found to be correlated with liver fat percentage. These results suggest that calculating the AT index may be a more meaningful clinical follow-up method for NAFLD in adolescents with obesity than evaluating ALT and AST levels separately.

Since the ALT level was more strongly correlated with the risk of NAFLD than the AT index according to the ROC curve analysis, a multivariate analysis was performed with the ALT level and other influential factors, such as the TyG index, HOMA-IR score and male sex, and an NAFLD probability scale was created to help clinicians diagnose NAFLD, with a total of 4 points, with each point increasing the risk of NAFLD by 3.36 times (95% CI: 2.11–5.88). This is a primitive probability scale first introduced in the diagnosis of NAFLD. Scales with higher diagnostic rates that clinicians can easily use in practice should be developed through more comprehensive studies on this subject. In studies with larger patient groups, this scale can be evaluated with the AT index rather than the ALT level.

Conventional US is a cost-effective, accessible, and noninvasive diagnostic tool for detecting NAFLD. It can indicate the severity of steatosis but cannot distinguish between steatosis, steatohepatitis and fibrosis. The accuracy of US may vary depending on the experience of the operator [41]. Elastography is also a noninvasive imaging method that facilitates the detection of both steatosis and fibrosis. It distinguishes simple steatosis from steatohepatitis [42]. However, it is not available in every clinic. MRI is the most accurate noninvasive diagnostic method for evaluating liver steatosis and fibrosis [43]. While US and computed tomography can reliably detect NAFLD when liver fat is > 20%, MRI can detect steatosis when it is 5% [44]. for NAFLD However, MRI is not cost-effective diagnosis. In this regard, studies conducted with MRI can produce realistic and reliable results on this issue, and with these results, more practical methods with a higher diagnostic rate can be found.

### Study strengths and limitations

This study included a selected population of adolescents with obesity diagnosed with NAFLD (+) or (-) using MRI. This is the first study to examine the BMI SDS, WC SDS, TPMI, Tg/HDL, AT index, TyG, and LAP indices together in an adolescent age group and provides the first data on the usefulness of the LAP index in adolescents with obesity. Additionally, this study is the first to create a probability scale that can be an effective and cost-free diagnostic method for the diagnosis of NAFLD in adolescents. The limited sample size is one of the limitations of this research. Future studies with larger sample sizes may reveal clearer findings on this subject. In addition, the presence of US findings along with MRI findings in imaging methods for the diagnosis of NAFLD could reveal important findings for comparison.

## Conclusion

In summary, the current study revealed that IR-related parameters were the most strongly associated factors in the presence of NAFLD. The use of the AT and TyG indices in NAFLD (+) adolescents with obesity was significantly greater than that of the ALT and Tg indices, respectively. In this first study using the LAP index to assess the presence of NAFLD in adolescents with obesity, the LAP index was greater in the NAFLD (+) group and showed a positive correlation with liver fat percentage. Among the 9 parameters evaluated together in this study, the TyG index and HOMA-IR were determined to be good indicators of the presence of NAFLD, and threshold values were recommended. Based on the study findings, a probability scale consisting of 4 parameters (including sex, ALT, HOMA-IR, and TyG) was developed, which can be used to diagnose NAFLD with good sensitivity and specificity, practically without incurring extra costs.

## Data Availability

No datasets were generated or analysed during the current study.
